# Prevalence and factors associated with anemia among adolescents in Uganda: a cross-sectional analysis of a national survey

**DOI:** 10.1186/s12889-026-28722-1

**Published:** 2026-08-05

**Authors:** Nakitta Jane, Pamela Kihembo, Bridget Kabanyoro, Flaviano Mugalula, Hellen Nansumba, Dathan M. Byonanebye

**Affiliations:** 1 National Health Laboratory and Diagnostic Services, Kampala, Uganda; 2 College of Health Sciences, Makerere University, Kampala, Uganda

**Keywords:** Anemia, Adolescents, Uganda national panel survey, Hemoglobin

## Abstract

**Background:**

Previous studies have reported high rates of anemia among adolescents in low- and middle-income countries (LMICs). It is unclear whether anemia rates have improved following temporal improvements in national investments by government and nutrition programs. The aim of this study was to establish the prevalence and factors associated with anemia among adolescents in a nationally representative survey.

**Methods:**

This was a cross-sectional study to determine the prevalence and predictors of anemia in adolescents aged 10–19 years. We analyzed data from the Nutrition Module of the 2018/2019 Uganda National Panel Survey (UNPS) and included 1063 adolescents with hemoglobin measurements who participated in the 2018/2019 survey. We determined the proportion of adolescents (and 95% confidence intervals, CIs) with anemia using WHO cutoffs. We used bivariable followed by multivariable Poisson regression model with robust variance to determine associated factors. QGIS software was used for spatial analysis.

**Results:**

Of the 119,148 participants in the 2018/2019 UNPS dataset, 24,856 were adolescents, 1063 of whom had hemoglobin measurements were included in the analysis. Overall, 172 adolescents **(**16.2%, CI 14%-19%) had anemia, 124(11.7%) of whom had mild anemia, 43(4.1%) had moderate anemia, and 5(0.5%) had severe anemia. The prevalence of anemia was 1.77 [aPR 1.83, CI 1.39–2.41] times high in males than in females. Adolescents aged 15–19 years were 1.99 times [aPR 1.97 CI 1.48–2.61)] more likely to be anemic than adolescents aged 10–14 years. *Helicobacter pylori* infection positivity increased the prevalence of anemia (aPR = 1.87, 95% CI = 1.35–2.58). There were no significant regional differences in anemia prevalence.

**Conclusion:**

The prevalence of anemia in adolescents remains high in Uganda and is possibly associated with *Helicobacter pylori* infection. Anemia was paradoxically more prevalent in males than in females suggesting a recent shift in burden. Anemia and *Helicobacter pylori* screening and prevention should be integrated into adolescent and school health programmes in Uganda and should equally target males and females.

**Supplementary Information:**

The online version contains supplementary material available at https://doi.org/10.1186/s12889-026-28722-1.

## Background

Despite the implementation of targeted anemia reduction interventions, anemia remains a leading cause of disability-adjusted life years (DALYs) lost among 15–19 years old adolescents [1].Over 1 billion adolescents are anemic globally [1]. The prevalence of adolescent anaemia in Africa was estimated at 45% over the last 20 years and has increased by about a third in resource limited countries [2]. In Uganda, the overall prevalence of anemia is not known however the burden in adolescent girls aged 15–19 years lies between 10 and 15% [3]. The vulnerability of adolescents to anemia is due to the physiological and physical changes that occur at this age [2, 4]. Rapid puberty growth with a sharp increase in fat mass, blood volume and red blood cell mass increase the need for iron for myoglobin in muscles and hemoglobin in blood [2]. This dramatic increase in iron requirement among adolescents peaks between the ages of 14–15 years for girls and one to two years later for boys [5]. The overall iron requirement increases two to three folds from a preadolescence level of approximately 0.7–0.9 mg Fe/day to as much as 1.37–1.88 mg Fe/day in adolescent boys and 1.40–3.27 mg Fe/day in adolescent girls [5]. Although adolescents of both sexes are among groups at risk for the development of anaemia, evidence from literature suggests that females are more vulnerable [2, 6]. Physiological losses such as loss of blood in case of heavy menstrual bleeding in adolescent females increase anaemia susceptibility in this group [7]. Teenage pregnancies also place double burden on the physically and physiologically immature body of girls which increases the likelihood of anaemia [7]. It is important to note that adolescent boys although an understudied group suffer from a high burden of malnutrition particularly thinness which predisposes them to anaemia [6]. Cumulatively, these studies highlight the complexity of the determinants of anaemia and the need for targeted nutrition interventions for adolescents. Determining factors include age, socio- economic status, dietary diversity and menstruation. Adolescents living in rural areas and those with low dietary diversity scores are more likely to be anemic [2]. This is possibly worsened by limited access to iron-rich fruits, vegetables and animal source foods as a consequence of food insecurity [8]. Additionally, factors such as stunting, malnutrition and parasitic infections, notably malaria and hookworm infestation, are often associated with increased anaemia prevalence [2].

Anemia has deleterious effects on adolescents, such as impaired cognition, poor school performance, impaired immunity and poor pregnancy outcomes [3, 9]. Additionally, anemia is associated with non-health-related consequences such as high health care expenditures and related socioeconomic problems in adolescents’ families and communities [10]. Among adolescents, anaemia affects not only the present health status but can also have deleterious effects in later life. The negative impacts at such a critical age of development can transcend adulthood and lead to higher risks [2].

In line with the WHO’s Global Nutrition Target calls for a 50% reduction in anemia prevalence among women of reproductive age (15–49 years) by 2030, several anemia reduction strategies, such as iron supplementation in pregnant women and adolescent girls, increased coverage for malaria control interventions and access to antiretroviral therapy, have been recommended to lower anemia prevalence [11]. In Uganda, the Ministry of Health has embarked on a number of multisectoral nutrition interventions, including the production of biofortified and iron-rich crops; the provision of iron, anthelmintic and vitamin A supplements to adolescents; family planning services; intensified malaria prevention and treatment; and the promotion of hygiene through increased access to water, improved latrines, hand washing, and infectious disease prevention [12].

Despite multisectoral efforts to reduce the burden of anemia among adolescents, little is known about whether the implemented programmes contribute to a reduction in anemia burden. This study aimed to the prevalence and factors associated with anemia among selected adolescents in Uganda using the data from a nationally representative survey.

## Methods

The study was a cross-sectional secondary analysis of the nutrition module of Uganda National Panel Survey, conducted from 2018 to 2019. The survey is conducted annually since 2009 and collects data on several indicators of nutritional and health status among children (10–14) years and adults (15–49) years. Data collection is conducted at two time points over a 12 months period to reduce respondent fatigue. Stratified random sampling combined with multi-stage sampling technique is deployed. Overall, the sampling allocation strives for reliable estimates of Kampala City, other Urban Areas, Central Rural, Eastern Rural, Western Rural, Northern Rural as well as Karamoja sub-region. In 2018/2019, the UNPS found 3,242 households eligible for the survey but only 3,176 households (*N* = 12,374) completed the full interview and were included in analysis [13]. The full interview consisted of the household roster, household questionnaire, woman questionnaire, agricultural questionnaire and the nutrition module. Data was collected on age in years, residence, region, dietary diversity and WASH (Water, sanitation and Hygiene) indicators. Blood was also collected to run the following assays; Hemoglobin test to assess anaemia status, malaria and *H. pylori* tests. Out of 12,374 participants that were recruited in the UNPS 2018/2019 survey, only 1078 were adolescents aged 10–19 years.15 out of 1078 adolescents had missing hemoglobin measurements and were excluded. Using Kish Leslie formular, the estimated sample size was 296 which increased to 653 after adjusting for design effect of 2 and non-response rate of 10%. However, since this was a secondary analysis of the Uganda National Panel Survey and the data was available, no sampling was done and all 1063 adolescents that met the inclusion criteria were included in the study.

### Outcome variable

The outcome variable of the study was anemia and this was defined as hemoglobin (Hb) concentrations below WHO age and gender standardized cutoffs as follows; Hb < 11.5 g/dL in adolescents aged 10–11 years, Hb < 12.0 g/dL in adolescents aged 12–14 years and non-pregnant female adolescents aged 15–19 years, Hb < 13.0 g/dL in male adolescents aged 15–19 years, Hb < 11.0 g/dL in pregnant adolescents (1st and 3rd trimesters) and Hb < 10.5 g/dL in pregnant adolescents (2nd trimester) [14]. Anaemia was also classified according to severity; mild, moderate and severe anaemia following WHO cutoffs which are both age and gender-specific. Anemia severity was classified according to haemoglobin concentration. Among participants aged 10–11 years, anemia was defined as mild (11.0–11.4 g/dL), moderate (8.0–10.9 g/dL), and severe (< 8.0 g/dL). For adolescents aged 12–14 years and females aged 15 years and above, anemia was classified as mild (11.0–11.9 g/dL), moderate (8.0–10.9 g/dL), and severe (< 8.0 g/dL). For males aged 15 years and above, mild anemia was defined as haemoglobin concentrations of 11.0–12.9 g/dL, moderate anemia as 8.0–10.9 g/dL, and severe anemia as < 8.0 g/dL [14, 15]. Hemoglobin results were adjusted for elevation but not smoking [13]. Anemia was analysed as a binary variable (yes/no).

### Exposure variables

The study included age, sex, residence, subregion, pregnancy, individual dietary diversity, malaria infection, *H. pylori* infection, pica syndrome, food consumption score, wealth index, presence of hand-washing facility, type of toilet used, use of a shared toilet facility and attendance of formal education as exposure variables. Data was collected via a structured questionnaire. Age was categorized into two age groups: (10–15) years and > 15 years. Gender was included as a binary variable (male/female). Residence was included as a binary variable (rural/urban). Current pregnancy status was included as a binary variable (yes/no). Dietary diversity was assessed by asking adolescents to describe their food and beverage consumption in the last 24 h before the survey for the purpose of calculating the Individual dietary diversity (IDDS). IDDS was defined as a count of the number of food groups out of 9 defined food groups an adolescent had consumed in the last 24 h preceding the survey [16]. The 9 food groups used to calculate the IDDS included i) starchy staples, ii)dark green leafy vegetables, iii)other vitamin A rich vegetables, iv) other fruits and vegetables, v)organ meat, vi)meat and fish, vii)eggs, viii) legumes, nuts and seeds and ix) milk and milk products [16]. IDDS ≥ 4 was categorized as high dietary diversity, whereas IDDS < 4 was classified as low dietary diversity on the basis of cutoffs used in another study performed to measure dietary diversity among adolescents in Uganda [17]. Malaria and *Helicobacter pylori* infections were analyzed as binary variables (positive/negative). A blood sample was collected by venipuncture using a butterfly needle into a vacutainer containing the anticoagulant ethylenediaminetetraacetic acid. Approximately 5–7 mls of blood were collected to test for malaria status, hemoglobin concentration and helicobacter pylori infection. The presence of malaria *Plasmodium falciparum* antibodies in blood was measured using a rapid test kit (RTK, CareStart Malaria Pf HRP2, Acces Bio, Inc, Somerest, NJ, USA). A positive malaria test indicated past or current malaria infection. The presence of Helicobacter pylori antibodies in blood was tested using a RTK (Hexagon Helicobacter pylori immunochromatographic rapid test kit, Human Diagnostics Worldwide, Wiesbaden, Germany). A positive Helicobacter pylori test indicated a past or current infection. Pica was defined as consumption of any dirt, earth, termite mounds, or clay from any source in the last 24 h preceding the survey. Participants were asked if they consumed any dirt, earth, termite mounds or clay from any source in the last 24 h before the survey. Pica was analyzed as a binary variable yes/no to indicate presence/absence. The food consumption score (FCS) was calculated and categorized as poor for scores (0–21), borderline for scores (21.5–35) and acceptable for scores (> 35) on the basis of WFP cutoffs [18]. Data on household food consumption and wealth index was captured at household level. The head of the household in which adolescent resides was asked about food and beverage consumption of the household both at home and away from home in the last seven days prior to the survey for the purpose of calculating the FCS. The household head was asked about the assets owned by all household members with the purpose of calculating the household’s wealth index. The wealth index was computed following the guidelines on the DHS program website and categorized as poorest, poorer, middle, richer and richest [19]. The presence of a handwashing facility, formal education attendance and use of a shared toilet were included as binary variables (yes/no). The type of toilet was categorized as unimproved toilet or improved toilet.

### Statistical analysis

Descriptive statistics were computed for the prevalence of anemia. We determined the proportion of adolescents with anemia (and 95% confidence intervals, Cis) according to known cutoffs: <11.5 g/dL in children aged 5–11 years, < 12.0 g/dL in children aged 12–14 years and non-pregnant female adolescents aged 15–19 years, < 13.0 g/dL in male adolescents aged 15–19 years, < 11.0 g/dL in pregnant adolescents (1st and 3rd trimesters) and < 10.5 g/dL in pregnant adolescents within the 2nd trimester. Candidate variables were selected based on the study conceptual framework and evidence from previous literature on determinants of adolescent anaemia. We used bivariable followed by multivariable Poisson regression model with robust variance to determine associated factors. Only candidate predictors of anemia with *P* ≤ 0.20 in bivariate analyses were included in the multivariable analysis. Multivariate analysis generated adjusted prevalence rate ratios with 95% confidence intervals. To identity collinearity, I computed for variance inflation factor (VIF) where VIF > 5 suggested collinearity. I also assessed for confounding at a 10% cutoff. I used backward elimination to address multicollinearity issues and determine model fitness of the final variable. Missing data were evaluated for all study variables. The proportion of missing observations for each variable is reported in Supplementary Table S1. Adolescents without haemoglobin test results were excluded from the analysis because the study outcome could not be ascertained. For explanatory variables with missing values, a separate “missing” category was created and included in both bivariate and multivariable analyses using the missing indicator method, thereby retaining participants with incomplete covariate data in the analytical sample. To assess for the spatial distribution of anaemia among adolescents, we computed anaemia prevalence rates among adolescents stratified to the 15 subregions in Uganda which included West Nile, Acholi, Karamoja, Bunyoro, Lango, Teso, Bugisu, Bukedi, Busoga, Central 2, Central 1, Kampala, Tooro, Ankole and Kigezi. QGIS software was then used to map this data across the subregions, applying a color gradient to represent varying value ranges for clearer visual interpretation on the generated chlorepath map.

## Results

Among the 1063 (55.5%) adolescents included in the analysis, 590 (55.5%) were females, 38 (6.4%) of whom were pregnant at the time of the survey. The median (IQR) age of the participants was 14.1 (11, 15) years. The study participants were distributed across the 4 regions, but the majority (78.7%, 837/1063) were from rural areas and had poor socioeconomic status. Additionally, 868 (81.7%) had poor food consumption, and 731 (68.8%) reported using unimproved toilet facilities. The rates of malaria and *Helicobacter pylori* infections were < 10%. (Table 1)

**Table 1:** Characteristics of study participants stratified by gender

^Variable^	^Male; (*n*=473)^	^Female (*n*=590)^	^Overall(*n*=1063)^
	^*n* (%)^	^*n* (%)^	^*n* (%)^
^Age^			
^ 10 –14 years^	^268(56.7)^	^312(52.9)^	^580(54.6)^
^ 15–19 years^	^205(43.3)^	^278(47.1)^	^483(45.4)^
^ Median (IQR) age^	^13.9 (2.8)^	^14.3 (2.9)^	^14 (12–17)^
^Residence^			
^ Rural^	^383(80.9)^	^454(76.9)^	^837(78.7)^
^ Urban^	^90 (19.0)^	^135(22.9)^	^225(21.2)^
^ Missing^		^1(0.2)^	^1 (0.01)^
^Pregnancy^			
^ Yes^	^0(0.0)^	^38(6.4)^	^38(6.4)^
^ No^	^257(100)^	^552(93.6)^	^552(93.6)^
^Region^			
^ Central^	^104(21.9)^	^139(23.6)^	^243(22.9)^
^ Eastern^	^136(28.8)^	^159(26.9)^	^295(27.8)^
^ Northern^	^118(24.9)^	^181(30.7)^	^299(28.1)^
^ Western^	^104(21.9)^	^106(17.9)^	^210(19.8)^
^ Missing^	^11(2.3)^	^5(0.9)^	^16(1.5)^
^Household food consumption^			
^ Poor food consumption^	^388(82.0)^	^480(81.4)^	^868(81.7)^
^ Borderline food consumption^	^66(13.9)^	^80(13.6)^	^146(13.7)^
^ Acceptable food consumption^	^19(4.0)^	^30(5.1)^	^49(4.6)^
^Handwashing facility^			
^ No^	^386(81.6)^	^473(80.2)^	^859(80.8)^
^ Yes^	^51(10.8)^	^67(11.4)^	^118(11.1)^
^ Missing^	^36 (7.6)^	^50(8.5)^	^86(8.1)^
^Toilet type^			
^ Improved toilet facility^	^130(27.5)^	^202(34.2)^	^332(31.2)^
^ Unimproved toilet facility^	^343(72.5)^	^388(65.8)^	^731(68.8)^
^Shared toilet facility^			
^ Yes^	^128(27.1)^	^146(24.8)^	^274(25.8)^
^ No^	^309(65.3)^	^394(66.8)^	^703(66.1)^
^ Missing^	^36(7.6)^	^50 (8.5)^	^86 (8.1)^
^Wealth Index^			
^ Poorest^	^78(16.5)^	^107(18.1)^	^185(17.4))^
^ Poorer^	^95(20.1)^	^110(18.6)^	^205(19.3)^
^ Middle^	^106(22.4)^	^93 (15.8)^	^199(18.7)^
^ Richer^	^89(18.8)^	^120(20.3)^	^209(19.7)^
^ Richest^	^63(13.3)^	^104(17.6)^	^167(15.7)^
^ Missing^	^42(8.9)^	^56(9.5)^	^98 (9.2)^
^Current/Past *H. pylori* infection^			
^ Positive^	^45(9.5)^	^57(9.7)^	^102(9.6)^
^ Negative or invalid^	^428(90.5)^	^533(90.3)^	^961(90.4)^
^Current malaria infection^			
^ Positive^	^33(6.9)^	^49(8.3)^	^82(7.7)^
^ Negative or invalid^	^440(93.0)^	^541(91.7)^	^981(92.3)^

Among the 1063 adolescents included in the analysis, 172 (16.2%, 95% CI 14%-19%) had anemia, 124 (11.7%) had mild anemia, 43 (4.1%) had moderate anemia, and 5 (0.5%) had severe anemia. The mean (SD) hemoglobin level was 13.4 (1.6) g/dL for both male and female participants. The overall prevalence of anemia in male adolescents (21.4%, 95% CI 17.9%-25.4%) was greater than that in female adolescents (12.0%, 95% CI 9.6%-14.9%). Among the 101 male adolescents with anemia, 79 (16.7%) had mild anemia, 21 (4.4%) had moderate anemia, and 1 (0.2%) was severely anemic. Among the 71 female adolescents with anemia, 45 (7.6%) had mild anemia, 22 (3.7%) had moderate anemia, and 4 (0.7%) were severely anemic (Table 2).

**Table 2:** Prevalence and severity classification of anemia based on sex

Variable	Male(*n* = 473)	Female(*n* = 590)	Overall(*n* = 1063)
Mean (SD) Hb (g/dL)	13.4(1.5)	13.4(1.7)	13.4(1.6)
Prevalence of anemia	*n *(%)	*n *(%)	*n* (%)
No anemia	372(78.7)	519(87.9)	891(83.8)
Anemia	101(21.4)	71(12.0)	172(16.2)
Anemia classification			
Mild anemia	79(16.7)	45(7.6)	124(11.7)
Moderate anemia	21(4.4)	22(3.7)	43(4.1)
Severe anemia	1(0.2)	4 (0.7)	5(0.5)

Age group, *H. pylori* infection status and sex were significantly associated with anemia in adolescents according to multivariate analysis. The prevalence rate of anemia was higher among adolescents aged 15–19 years than among those aged 10–14 years (aPR = 1.97, 95% CI = 1.48–2.61). The prevalence rate of anemia was also higher in male adolescents than in female adolescents (aPR = 1.83, 95% CI 1.39, 2.41). Adolescents who tested positive for *H. pylori* infection were more likely to have anemia (aPR = 1.87, 95% CI 1.35, 2.58) (Table 3). 

**Table 3:** Factors associated with anaemia at bivariate and multivariate analysis

Variable	No anemia *N* (%)	Anemia *N* (%)	cPR (95% CI)	*P* - value	aPR (95% CI)	*P* value
Age group						
10–14 years	514(88.6)	66(11.4)	Reference	Reference		
15–19 years	377(78.1)	106(21.9)	**1.99(1.45**,**2.56)**	< 0.001*	**1.97(1.48**,**2.61)**	< 0.001**
Sex						
Female	519(87.9)	71(12.0)	Reference		Reference	
Male	372(78.7)	101(21.4)	**1.77(1.34**,**2.34)**	< 0.001*	**1.83(1.39**,**2.41)**	< 0.001**
Residence						
Rural	702(83.9)	135(16.1)	Reference			
Urban	188(83.6)	37(16.4)	1.02(0.73,1.42)	0.909		
Missing	1 (100.0)					
Region						
Central	212(87.2)	31(12.8)	Reference		Reference	
Eastern	242(82.0)	53(17.9)	**1.41(0.94**,**2.12)**	**0.101**	1.34(0.90,1.99)	0.146
Northern	251(83.9)	48(16.1)	1.26(0.83,1.91)	0.282	1.22(0.81,1.83)	0.332
Western	176(83.8)	34(16.2)	1.27(0.81,1.99)	0.300	1.25(0.81,1.94)	0.316
Missing	10(62.5)	6(37.5)				
Current Pregnancy? (females only)						
Yes	36(94.7)	2(5.2)	1.27(0.33,4.85)	0.723		
No	483(87.5)	69 (12.5)	Reference			
Current Malaria infection						
Positive	64(78.1)	18(21.9)	**1.40(0.91**,**2.16)**	0.129*	1.23(0.79,1.89)	0.349
Negative or invalid	827(84.3)	254(15.7)	Reference		Reference	
Current/Past *H. pylori* infection						
Positive	73(71.6)	29(28.4)	**1.91(1.35**,**2.69)**	<0.001*	**1.87(1.35**,**2.58)**	<0.001**
Negative or invalid	818(85.1)	143(14.9)	Reference		Reference	
Individual dietary diversity						
Low diet diversity	582(83.5)	115(16.5)	Reference			
High diet diversity	80(80.0)	20(20.0)	1.21(0.79,1.86)	0.376		
Missing	229(86.1)	37(13.9)	0.16(0.14,0.19)			
Household Food consumption score						
Poor	738(85.0)	130(15.0)	Reference		Reference	
Borderline	115(78.8)	31(21.2)	**1.42(0.99**,**2.01)**	0.051*	1.38(0.99,1.93)	0.058
Acceptable	38(77.6)	11(22.5)	**1.49(0.87**,**2.58)**	0.145*	1.48(0.87,2.52)	0.143
Pica syndrome						
No	627(84.2)	118(15.8)	Reference			
yes	264(83.0)	54(16.9)	1.07(0.79,1.45)	0.643		
Receipt of deworming drugs						
Yes	229(84.8)	41(15.2)	Reference			
No or don’t know	662(83.5)	131(16.5)	1.09(0.79,1.50)	0.609		
Receipt of preventive malaria drugs						
Yes	269(81.5)	61(18.5)	Reference			
No	208(84.2)	39(15.8)	0.85(0.59,1.23)	0.400		
Missing	414(85.2)	72(14.8)				
Handwashing facility						
No	729(84.9)	130(15.1)	1.19(0.72,1.96)	0.493		
Yes	103(87.3)	15(12.7)	Reference			
Missing	59(68.6)	27(31.4)	0.13(0.08,0.20)			
Toilet type						
Improved toilet facility	288(86.8)	44(13.3)	Reference		Reference	
Unimproved toilet facility	603(82.5)	128(17.5)	**1.32(0.96**,** 1.81)**	0.085*	1.27(0.93,1.72)	0.129
Shared toilet						
Yes	227(82.9)	47(17.2)	1.23(0.89,1.69)	0.202		
No	605(86.1)	98(13.9)	Reference			
Wealth Index						
Poorest	159(85.9)	26(14.1)	0.94 (0.56,1.56)	0.807		
Poorer	175(85.4)	30(14.6)	0.98(0.59,1.59)	0.928		
Middle	160(80.9)	38(19.1)	1.28(0.80,2.02)	0.301		
Richer	184(88.0)	25(11.9)	0.79(0.48,1.34)	0.394		
Richest	142(85.0)	25(14.9)	Reference			
Missing	70(71.4)	28(28.6)	0.15(0.10,0.21)			
Formal school attendance						
Yes	151(82.5)	32(17.5)	Reference			
No	740(84.1)	140(15.9)	0.91(0.64,1.29)	0.596		

*P values < 0.2 significant at bivariate analysis. **P values <0.001 significant at multivariate analysis

**Fig. 1:**
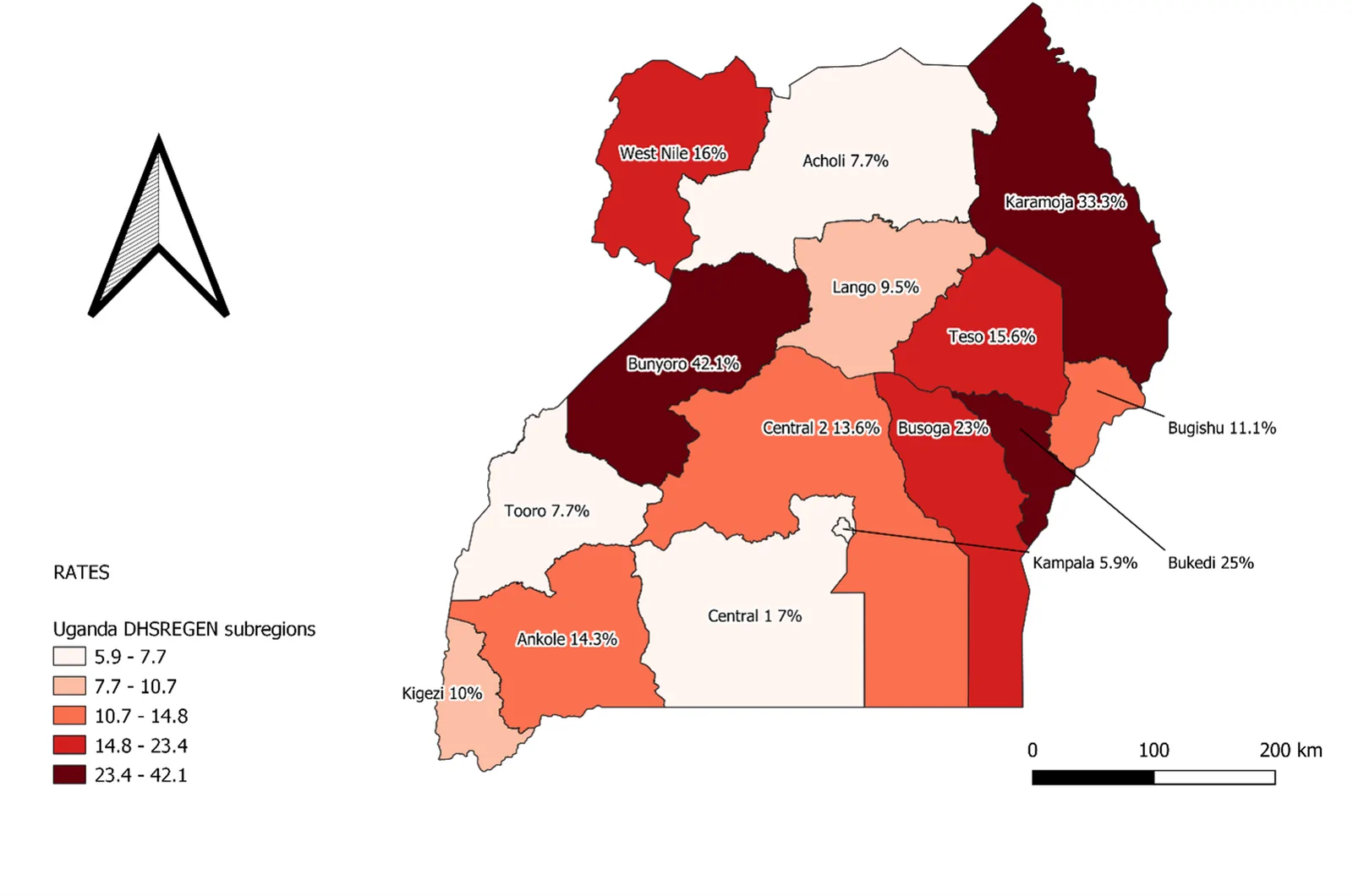
A chlorepath map showing percentage prevalence rates of anaemia among adolescents in Uganda stratified by subregion

The Karamoja, Bunyoro and Bukedi subregions presented the highest prevalence rates, at 33.3%, 42.1% and 25%, respectively. The Acholi, Tooro, Central 1 and Kampala subregions had anemia prevalence rates lower than 7.7%. The prevalence of anemia did not differ across the four major regions, i.e., northern, southern, central and eastern, according to multivariate analysis (P values > 0.05). (Fig. 1)

## Discussion

In this cross-sectional study to determine the prevalence and factors associated with anemia among adolescents in a nationally representative survey, the overall prevalence of anemia was 16.2%. The factors associated with anemia were older age (15–19 years), *Helicobacter pylori* infection positivity and male sex. The Bunyoro subregion in eastern Uganda had the highest anemia incidence (42%), followed by the Karamoja subregion in northeastern Uganda (33%). The Central Region had the lowest anemia prevalence (7%). The prevalence in this study was comparable to the burden reported among women aged 15–45 years in the UNPS 2018/2019 survey (16.7%), from which these adolescent data were extracted [13], although it was lower than the prevalence of 26% reported in the most recent UNPS survey in 2019/2020 [20]. The prevalence in this study was also comparable to that reported in a similar study conducted among adolescents aged 15–19 years in a national survey, UDHS 2018/2019, which reported a prevalence between 10 and 15% [3]. The anemia prevalence in adolescents reported in this study was comparable to the burden in the general population, implying that anemia prevention programmes targeting adolescents have been successful and should continue. The majority of the adolescent anemia cases in this study were mild (12.3%) compared with moderate and severe cases, which is comparable to the findings of other studies performed in similar contexts [21–23]. This was expected since this was a household survey and not a hospital-based study. According to the WHO classification for public health significance, anemia prevalence rates of 5.0–19.9% are categorized as having mild public health significance [24]. This study revealed that the prevalence of anemia among adolescents in Uganda is of mild public health significance, which calls for proactive measures such as public health education to prevent escalation of the problem to moderate/severe public health significance.

This study revealed a statistically significant association between sex and anaemia among adolescents. The prevalence of anaemia was higher among male adolescents (21.4%) than among female adolescents (12.0%). Although unexpected, this was consistent with findings from comparative studies conducted among adolescents in Ethiopia, Sudan and Tanzania [22, 23]. Since adolescent girls are commonly reported to have a higher burden of anaemia due to menstrual blood loss, the higher prevalence observed among boys in this study may partly reflect differences in social and environmental characteristics. A greater proportion of boys resided in rural areas (80.9% versus 76.9% among girls) and lived in households with unimproved sanitation facilities (72.5% versus 65.8%). These conditions may increase exposure to infections and other environmental factors that contribute to anaemia. Boys were also slightly less likely to belong to the richest wealth category (13.3% versus 17.6%) and slightly less likely to live in households with acceptable food consumption (4.0% versus 5.1%), suggesting modest socioeconomic and dietary disadvantages. However, the differences in these characteristics were relatively small and are unlikely to fully account for the nearly twofold difference in anaemia prevalence observed between boys and girls. Furthermore, the study assessed individual dietary diversity and adjusted for several important covariates including socioeconomic status, malaria infection and H. pylori infection. Notably, the prevalence of malaria infection and H. pylori infection was similar between boys and girls, suggesting that these measured factors do not adequately explain the observed sex disparity. The finding may therefore reflect factors that were not captured in the survey. For example, adolescent boys experience rapid growth and expansion of lean body mass during puberty, processes that substantially increase iron requirements. If these increased physiological demands are not matched by adequate iron intake, boys may be at increased risk of anaemia despite the absence of menstrual blood loss. Residual confounding may also have contributed to the findings. The survey did not collect information on several determinants of anaemia, including iron intake, helminth infections, inflammatory conditions, inherited hemoglobin disorders and pubertal stage that may differ by sex and influence hemoglobin concentrations. It should also be considered that anaemia was defined using hemoglobin concentration alone, and sex-specific hemoglobin thresholds were applied according to WHO recommendations. Because older adolescent boys are classified using a higher hemoglobin cut-off than girls, some boys with hemoglobin concentrations near the diagnostic threshold may have been more likely to be classified as anemic. This may have contributed, at least in part, to the higher prevalence observed among boys. The reasons underlying the higher prevalence of anaemia among boys therefore remain incompletely understood and warrant further investigation. Future studies incorporating biomarkers of iron status and inflammation, detailed dietary assessments and measures of pubertal development may help clarify the mechanisms underlying these sex differences. In this study, older adolescents (15–19 years) were more likely to be anemic (aPR = 1.97, 95% CI 1.48–2.61) than younger adolescents (10–14 years), which is in agreement with the findings of a study conducted in Sudan [23]. This could be due to inadequate dietary intake since adolescents in the 15–19 years age group are often more independent in choosing their diets, which may be less balanced and lacking essential nutrients such as iron, folic acid, and vitamin B12, which can lead to nutrient deficiencies that contribute to anemia. This finding implies that dietary modifications targeting older adolescents (15–19 years), including male and nonpregnant females, could minimize nutrient deficiency and lower anemia prevalence. A quasi-experimental study performed by the Uganda Multisectoral Food Security and Nutrition Project revealed that anemia prevalence was lower in the adolescent experimental group (26%), which received an intervention of increased consumption of micronutrient-rich food, than in the nonexperimental group (36%) after a follow-up period of 5 years [25].

Similar to several studies, this study revealed that *Helicobacter pylori* infection was high (almost 10%) and thus increased the prevalence of anemia among adolescents [26]. Malaria infection positivity also increased anemia incidence; however, the association was not significant. This finding suggests that malaria control measures are effective and that more efforts should instead be directed towards *Helicobacter pylori* eradication programs and improvements in water, sanitation and hygiene infrastructure as measures to reduce the burden of anemia. 

Contrary to previous studies, this study suggested a positive association between high dietary diversity and anaemia prevalence. A study conducted among adolescents in Ethiopia, Sudan, and Tanzania reported that higher dietary diversity reduced the risk of anaemia [23]. In contrast, adolescents with adequate dietary diversity in our study were 1.21 times more likely to be anemic. This unexpected finding suggests that dietary diversity alone may not fully capture the nutritional factors influencing anaemia risk, particularly the adequacy, bioavailability and absorption of key nutrients such as iron. One possible explanation is that dietary diversity reflects the number of food groups consumed but does not provide information on the quantity of nutrients obtained or the bioavailability of iron from consumed foods. The majority of participants in this study lived in rural areas (837/1063, 78.7%), where diets may predominantly rely on plant-based foods. Although such diets may contain multiple food groups, they often provide mainly non-heme iron, which is absorbed less efficiently compared with heme iron from animal-source foods. Therefore, adolescents may meet dietary diversity criteria while still having inadequate intake of bioavailable iron contributing to anaemia risk. Furthermore, household food consumption characteristics in this study indicate widespread underlying food access challenges, with 81.7% of adolescents living in households classified as having poor food consumption. Thus, higher individual dietary diversity may represent access to a wider range of foods on the day of assessment but may not necessarily indicate consistent access to nutrient-dense foods required to meet adolescent nutritional requirements. This is particularly important during adolescence, a period characterized by rapid growth, increased lean body mass development and increased micronutrient requirements. If dietary intake does not sufficiently meet these increased physiological demands, adolescents may remain vulnerable to anaemia despite consuming a diverse diet. Additionally, the findings should be interpreted considering the potential influence of missing dietary diversity data. Approximately 25% of participants had missing information on dietary diversity although Missing Indicator Method was used to handle the missingness. If missingness was associated with dietary patterns or anaemia status, the observed association may have been affected. However, the analytical sample size (*n* = 797) remained above the minimum required sample size for this objective, maintaining adequate statistical power. These findings highlight the importance of looking beyond dietary diversity as a standalone indicator when examining nutritional determinants of anemia among adolescents in Uganda. Future studies should incorporate more comprehensive dietary assessments including iron intake, consumption of animal-source foods, dietary inhibitors and enhancers of iron absorption alongside biomarkers of iron status and inflammation to better understand the complex relationship between diet and anaemia among adolescents. This study revealed no significant regional differences in anemia incidence via multivariate analysis. The prevalence of anemia among adolescents was greater in the Bukedi (25%), Busoga (23%),Teso (15.6%) and Karamoja (33.3%) subregions (Crude PR = 1.41, 95% CI = 0.94, 2.12), which is comparable to the findings of a study performed in Uganda [27]. The high prevalence reported in Eastern region, particularly in the Bukedi subregion, could be due to poor dietary intake. In many parts of Eastern Uganda, particularly in rural areas, people consume diets that are low in iron, such as “emboli” (sweet potatoes and beans), as staples, which could contribute to the high prevalence. The high anemia prevalence in the Karamoja subregion could be due to food insecurity resulting from poor crop harvests given the harsh climatic conditions (semiarid). Surprisingly, this study revealed a low anemia prevalence in the Acholi subregion, which could be because of favorable climatic conditions (tropical savannah) that support local farming practices, hence allowing access to a variety of foods in this region, which could contribute to the lower prevalence. Additionally, UDHS 2022 showed an improvement in health indices within northern Uganda, as evidenced by a decline in neonatal and under 5 child mortality rates to 45% in the Acholi subregion [28].The prevalence of anemia among adolescents in Bunyoro (42.1%) was also high, which was comparable to the findings of an infant study [27]. This finding could be due to high malnutrition rates and the influx of refugees from South Sudan and the Democratic Republic of the Congo. The Central 1 subregion had the lowest anemia prevalence (7%), as expected, since it includes the capital city, Kampala, and therefore has improved infrastructure and higher socioeconomic status. These findings suggest that strategies directed toward food-based interventions such as fortification and food assistance programs can contribute to reducing the prevalence of anemia among adolescents in Uganda.

Although some adolescents were pregnant, it is unlikely that this introduced major bias for several reasons. First, the hemoglobin cutoff for anemia was lower for pregnant women, which is consistent with the WHO diagnostic criteria. Adjusting for pregnancy status as a confounder would amount to double adjustment and lead to misleading estimates. Second, the proportion of adolescents who were pregnant was low (3.6%). Third, the rates of anemia in pregnant adolescents were also low (5.2%), suggesting that the number of anemic adolescents who were also pregnant is expectedly low to affect the results. Despite the above, there is residual bias due to the nature of the design. There were no data on antenatal care attendance or other anemia prevention interventions, such as the receipt of iron and folic acid supplements.

This study has several limitations. Owing to the cross-sectional study design, we were unable to establish causality between candidate predictors and anemia status; however, our study contributes to the limited evidence base in this understudied population group (adolescents). Additionally, the use of a secondary dataset (UNPS) limits the variables available for use. For example, this study did not explore how multiple biomarkers of nutritional status, such as vitamin B12, serum ferritin and other potentially important biomarkers, could influence anemia status. However, the inclusion of variables related to individual dietary diversity and household food consumption scores provides a proxy for an individual’s nutritional status. Although the UNPS employed a complex survey design, sampling weights and survey design features were not incorporated into the present analysis, which may limit the national representativeness of the estimates.Missing covariate data were handled using the missing indicator method but additional sensitivity analyses or multiple imputation were not done; therefore, findings should be interpreted with consideration of the assumptions underlying this approach. However, this study also has several strengths. This study was a nested analysis of a nationally representative survey and therefore was very well powered. The study investigated the association between infections such as *Helicobacter pylori* which will become an important risk factor for anemia as malaria prevention and control strategies are scaled up. Furthermore, this study investigated the grades of anemia according to severity. This study also demonstrated the geospatial distribution of the anemia burden among adolescents in Uganda, which has not been explored in most anemia studies.

## Conclusion

The prevalence of adolescent anemia in Uganda is a mild public health concern, with a greater anemia burden in males, older adolescents aged 15–19 years and adolescents with *H. pylori* infection. The implementation of proactive measures, especially in schools, such as Helicobacter pylori screening, treatment and prevention through improved sanitation/hygiene in communities, can counteract the progression of anemia to severe/moderate public health significance.

## Supplementary Information

Supplementary Material 1.

## Data Availability

The study utilized a secondary source of data that is available on request and is available in the public domain through [https://microdata.worldbank.org/index.php/catalog/3795](.) .

## Abbreviations

DALYs: Disability Adjusted Life Years
SSA: Sub-Saharan Africa
WHO: World Health Organization
UDHS: Uganda Demographic Health Survey
UNPS: Uganda National Panel Survey
UBOS: Uganda Bureau of Statistics
Hb: Hemoglobin
IDDS: Individual Dietary Diversity Score
WFP: World Food Programme
FCS: Food Consumption Score
FAO: Food and Agriculture Organization
