# Supplementary material for: Prevalence and factors associated with anemia among adolescents in Uganda: a cross-sectional analysis of a national survey
Source: BMC Public Health. 2026 Aug 5;26:3164. doi: 10.1186/s12889-026-28722-1 (PMC13725981; doi:10.1186/s12889-026-28722-1)
Supplement: Supplementary file 1 [file 12889_2026_28722_MOESM1_ESM.docx]

| Variable | Missing n | Missing % |
| --- | --- | --- |
| Age | 0 | 0.00% |
| Residence | 1 | 0.01% |
| Pregnancy | 0 | 0.00% |
| Region | 16 | 1.50% |
| Household food consumption | 49 | 4.60% |
| Handwashing facility | 86 | 8.10% |
| Toilet type | 0 | 0.00% |
| Shared toilet facility | 86 | 8.10% |
| Wealth index | 98 | 9.2% |
| Current Malaria infection | 0 | 0.00% |
| Current/ Past H.pylori infection | 0 | 0.00% |
| Individual Dietary Diversity | 266 | 25.00% |
| Pica syndrome | 0 | 0.00% |
| Receipt of deworming drugs | 0 | 0.00% |
| Receipt of preventive malaria drugs | 0 | 0.00% |
| Formal school attendance | 0 | 0.00% |

**Supplementary Table S1 : Extent of missing data for variables included in the analysis**
